# The Role of Probiotics in Modulating the Gut Microbiome in Alzheimer’s Disease: A Review

**DOI:** 10.3390/foods14091531

**Published:** 2025-04-27

**Authors:** Yushi Dong, Xilin Wu, Yumeng Zhang, Adi Hu, Qian Zhou, Xiqing Yue, Zhenmin Liu, Mohan Li

**Affiliations:** 1 State Key Laboratory of Dairy Biotechnology, Shanghai Engineering Research Center of Dairy Biotechnology, Shanghai 200436, China; 2 College of Food Science, Shenyang Agricultural University, Shenyang 110866, China; 3 School of Life Sciences & Biotechnology, Shanghai Jiao Tong University, Shanghai 200240, China; 4 Liaoning Industrial and Information Technology Development Research Institute, Shenyang 110180, China

**Keywords:** probiotics, Alzheimer’s disease, gut microbiome

## Abstract

Alzheimer’s disease (AD) has emerged as a global public health priority characterized by escalating prevalence and the limited efficacy of current therapeutic approaches. Although the pathological complexity of AD is well-recognized, its underlying etiology remains incompletely elucidated. Current research highlights a bidirectional gut–brain axis (GBA) interaction, wherein gut microbiome perturbations may impair intestinal barrier stability, influence immune responses, and blood–brain barrier permeability through microbial metabolite-mediated pathways, thereby contributing to AD pathophysiology. Notably, probiotics demonstrate therapeutic potential by restoring gut microbiome homeostasis, reinforcing intestinal barrier integrity, and mitigating neuroinflammatory responses via GBA. This review focuses on investigating the gut microbiome alterations in AD pathogenesis, the interaction of probiotics with GBA, and its significance in AD pathogenesis. By synthesizing current clinical evidence, this review aims to establish a scientific foundation for probiotic-based interventions as a novel therapeutic strategy in AD management.

## 1. Introduction

Alzheimer’s disease (AD) is a progressive neurodegenerative disorder characterized by the gradual deterioration of cognitive, memory, and motor functions, with primary incidence in adults over 65 years [1]. Approximately 50 million people worldwide have dementia and AD accounts for approximately 70% of cases worldwide. According to World Health Organization statistics [2], a new dementia diagnosis occurs every three seconds worldwide. Projections indicate that the dementia population will rise to 139 million by 2050 due to aging demographics. Economically, dementia-related costs reached USD 1.3 trillion in 2019, with estimates suggesting this figure will climb to USD 2.8 trillion by 2030. These escalating trends pose significant challenges to global healthcare systems, public health, and socioeconomic structures.

Amyloid-β plaques and Tau protein neurofibrillary tangles (NFTs) are two typical pathological features of AD [3], which might lead to neuroinflammation, calcium homeostasis disturbance, excess iron accumulation, and oxidative stress, eventually contributing to neuronal cell death and then cognitive dysfunction [4,5]. Despite these established associations, the conclusive mechanism of these pathological hallmarks of AD remains undefined. Current therapeutic approaches primarily address symptom management rather than targeting disease progression, highlighting the critical need for multidimensional re-evaluation of AD pathogenesis.

Cognitive dysfunction represents the primary symptom of AD. Recent scientific attention has focused on the potential connection between the gut microbiome and cognitive function. Probiotics can directly interact with the host and gut microbiome, thus producing intestinal effects, such as enhancing the epithelial barrier function, modulating the systemic metabolic responses, and signaling with the central nervous system (CNS) [6]. The prebiotic effect is achieved by providing substrates for specific intestinal microbial groups. Prebiotics can promote their growth and metabolism, thus indirectly affecting intestinal function, the immune system, and nervous signals [7]. Several reports have illustrated that probiotics may delay neurodegenerative processes through cognitive function preservation and neuroinflammation suppression [8,9,10]. Specifically, probiotics regulate complex metabolic and biochemical processes of the gut microbiome to enhance central nervous system (CNS) function, facilitating gut–brain communication. This interaction between the enteric nervous system (ENS) and CNS occurs via the bidirectional gut–brain axis (GBA) [11]. Probiotics deliver various gut secretions through the GBA to inhibit inflammatory reactions, strengthen the immune system, and protect neuronal integrity. These discoveries reinforce the gut–brain interconnection while proposing novel therapeutic strategies for AD.

This review comprehensively analyzes gut microbiome–brain interactions, focusing on probiotic modulation of GBA-mediated AD pathology to facilitate the establishment of probiotic-based approaches for AD prevention and treatment.

## 2. Overview of AD

### 2.1. Pathogenesis of AD

AD is the most common type of dementia; however, its specific cause has not yet been identified. Moreover, effective care and treatment cannot be provided, even if AD is diagnosed. Currently, the pathogenesis of AD includes abnormal amyloid proteins, Tau protein phosphorylation-induced NFTs, neuroinflammation, neuroactive substance disorder, mitochondrial dysfunction, iron accumulation-induced ferroptosis, and oxidative stress [12]. Among them, amyloid-beta peptide (Aβ) aggregates and NFTs are the most widely studied. The essential precursor of Aβ plaque is Aβ precursor protein (APP), which is a type 1 transmembrane glycoprotein. Their functions are associated with neuronal synaptogenesis and plasticity. Two pathways are involved in the proteolytic metabolism of APP. The α-secretase cleavage pathway will not produce Aβ deposits, while the β-secretase enzyme sunders APP to neurotoxin formation and β-40 to 42 amino acid proteins [13]. Moreover, beta-amyloid is cleaved by β-secretase to induce APP accumulation. Aggregation of Aβ plaques in the brain stimulates mitogen-activated protein kinase and glycogen synthase kinase-3β signaling to induce hyperphosphorylation and polymerization of the Tau protein into insoluble NFTs [14].

### 2.2. AD, a Metabolic Syndrome?

The metabolic axis of Alzheimer’s disease pathophysiology involves dysregulated Aβ/Tau proteostasis, glia-driven neuroinflammatory cascades, mitochondrial dysfunction, ferroptosis, and oxidative stress pathways, which synergistically propagate a self-sustaining cycle of neurodegenerative progression [3,4,5].

**Figure 1:**
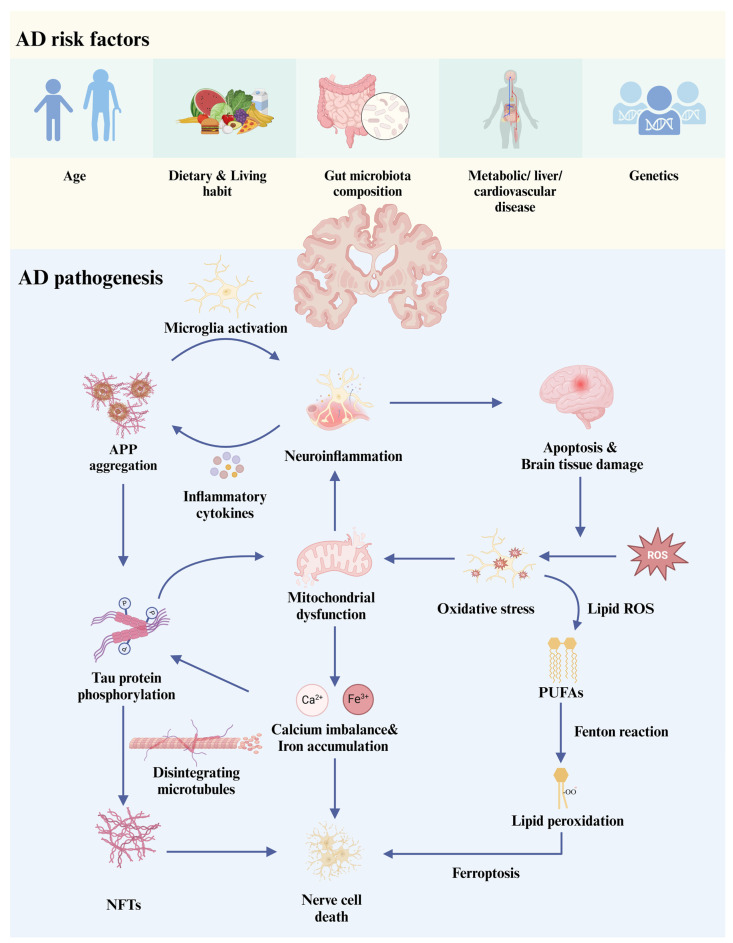
AD risk factors and the correlation between various pathologies of AD. AD, Alzheimer’s disease; APP, Aβ precursor protein; NFT, neurofibrillary tangle; ROS, reactive oxygen species; PUFAs, polyunsaturated fatty acids.

Excessive accumulation of the Aβ peptide promotes microglia activation, which leads to neuroinflammation [15]. Microglia and astrocytes participate in the innate immune function of the CNS and play a major role in the neuroinflammatory reactions in AD [16]. Microglia can clear up excess Aβ peptide through LDL receptor-related protein-1 [17]. However, excessive Aβ deposition activates microglia. Lipopolysaccharides (LPSs) can induce activated cells (M1-type microglia) to secrete inflammatory factors, eventually leading to an inflammatory reaction [18]. In addition, astrocytes can strengthen the stability of the blood–brain barrier (BBB). However, activated astrocytes will increase the permeability of the BBB, and more Aβ plaques will pass through the BBB to increase the production of pro-inflammatory factors [19]. This enhances the development of neuroinflammation, leading to a vicious cycle (Figure 1). However, the causal relationship between neuroinflammation and AD pathogenesis remains unclear. In a randomized double-blind study by Aisen et al. [20], patients with AD were treated with anti-inflammatory agents. The results showed that these two anti-inflammatory drugs did not reduce the cognitive ability of patients with AD. Although the Aβ abnormality in the brain is one of the important symptoms of AD, the underlying cause and molecular mechanism behind this process are not completely known. Clinical drug therapy for Aβ has not significantly reversed the cognitive decline related to AD [21]. These results suggest that a single element, such as the Aβ plaque or inflammatory factors alone, may not be enough to cause AD and other comprehensive factors may be involved.

Tau is a microtubule-associated protein that regulates axonal transport, elongation, and maturation and the maintenance of DNA and RNA integrity of neurons [22]. The aggregation of the Aβ peptide and Tau protein in the cortex and brain stem is the first pathological feature observed in the development of AD [23]. Aβ plaque in neurons will activate glycogen synthase kinase 3-β (GSK-3β), which can phosphorylate Tau protein. This wild-type protein breaks away from neural microtubules and spreads among neurons via a trans-synaptic mechanism, after which NFTs are deposited in the brain, leading to neuronal death [24]. An imbalance of calcium ions in the mitochondria is a key catalytic factor in this process. Overloading of calcium ions aggravates the hyperphosphorylation of the Tau protein, causing an imbalance in mitochondrial transport function and leading to oxidative stress [16]. The brain requires more dynamic energy than other tissues and is vulnerable to oxidative stress because of the defenseless antioxidant status of neurons [25]. Excessive iron accumulation has also been observed in the brains of patients with AD [5,26]. Iron overload can lead to hydroxyl radicals, ROS generation, and oxidative stress through the Fenton reaction, thereby destroying lipids, DNA, RNA, and proteins [27]. In turn, oxidative stress can enhance the acceleration of Aβ plaque and NFT formation. The process described above is illustrated in Figure 1.

### 2.3. Risk Factors in AD

Age is a significant risk factor for AD, apart from lifestyle (e.g., smoking and unhealthy diet), genetic inheritance, health status (e.g., diabetes, cardiovascular disease, and weakened immunity), and environmental factors (e.g., pollution and lack of education). [28] (Figure 1). Overall, the pathological mechanism of AD is not caused by a single element but may be the result of the interaction of various complex mechanisms. AD is a complex metabolic syndrome, and different genetic and environmental risk factors have comprehensive effects on the treatment of AD. Therefore, it is necessary to comprehensively explore new treatment strategies for AD from a new perspective. A complex relationship exists between the intestinal microbiome and human health, which influences health benefits by manipulating metabolism, the host immune system, and neurobehavioral traits [29]. Thus, the intestine regulates and participates in various metabolic mechanisms in different ways, playing a comprehensive role in human health.

## 3. The Gut Microbiome

### 3.1. Gut Microbiome Composition

The types and numbers of the intestinal microbiome are distributed across different areas of the human GI tract. The colon is the habitat of most microorganisms, while a relatively small number of microorganisms live in the stomach and small intestine [30,31,32]. Bacteria were the most abundant microbial species, accounting for 60%, and a few species included viruses, fungi, and archaea [29,33]. The GI tract in humans contains approximately 100 trillion microorganisms known as the gut microbiome, which are categorized into over 1500 species. The most dominant phyla were *Bacteroidetes*, followed by *Firmicutes*, *Proteobacteria*, *Fusobacteria*, *Tenericutes*, *Actinobacteria*, and *Verrucomicrobia* [34]. They produce thousands of metabolites that participate in host metabolic processes and defense mechanisms, thus influencing human health and function. In addition to helping the GI tract digest and absorb various substances and nutrients, protecting human beings from viruses, many diseases are related to intestinal microbiome dysfunction (such as inflammatory bowel disease [IBD], type 2 diabetes, obesity, cardiovascular diseases, AD, and depression) [35,36,37,38].

### 3.2. Role and Funciton

#### 3.2.1. Immunomodulatory and Metabolic Properties

The homeostasis of intestinal microorganisms is essential for the gut mucosal barrier and host immunity. The integrity of the gut mucosal barrier provides a favorable environment for metabolic processes. *Bifidobacterium breve* and *Lactobacillus salivarius* are the two main bacteria associated with stable immune system stability [39,40]. They can prevent auto-immune diseases (such as GI infection, diarrhea, and IBD) and allergic reactions by enhancing the secretion of some anti-bacterial substances (such as bacteriocins, H_2_O_2_, and organic acids) and host immune cells (such as macrophages and lymphocytes) [41,42]. Microorganisms facilitate nutrient digestion and absorption in the gut and can metabolize bioactive components, such as vitamins, to promote health [43,44]. Most vitamins cannot be synthesized in the human body and must be obtained externally. Some intestinal microorganisms are capable of de novo vitamin synthesis and supplementation [45]. Bifidobacteria promote the synthesis of fat-soluble vitamin K and most water-soluble vitamin B groups [46], including thiamine, riboflavin, pantothenic acids, folic acid, nicotinic acid, biotin, pyridoxine, and cobalamin.

#### 3.2.2. Protective Role in Various Diseases

Some intestinal microbes participate in the fermentation of indigestible carbohydrates (such as cellulose, hemicellulose, resistant starch, and lignin), which are the main metabolic functions of colon microorganisms [29,43,47,48]. This process produces short-chain fatty acids (SCFA), including acetate, propionate, and butyrate from Bacteroidetes and Firmicutes. These SCFA metabolites participate in the production and metabolism of bile acids, cholesterol, and fat by secreting glucagon-like peptide-1 (GLP-1) and peptide YY (PYY) [49]. They suppress appetite and ameliorate diet-induced obesity and hyperglycemia [50,51]. GLP-1 and PYY can be used as neuroprotective agents to inhibit the formation of Aβ plaque, exert anti-inflammatory effects, and mitigate oxidative stress [52,53]. SCFAs induce the secretion of insulin-like growth factor 1 (IGF-1) during cellulose fermentation [54]. IGF-1 promotes the secretion of calcium ions and mediates the osteoclast–osteoblast pathway to facilitate the growth, formation, and even remodeling of bones, ultimately diminishing the risk of osteoporosis and fracture [55]. Similar results were reported by Parvaneh et al. [56], where microorganisms, such as *Lactobacillus acidophilus* and *Lactobacillus plantarum* in the gut microbiome, increased bone density and remodeled bones by upregulating the expression of runt-related transcription factor 2 (Runx2) and bone morphometric protein 2 (Bmp2). *L. acidophilus* has been widely used as a probiotic supplement in commercial and medical fields, such as dairy processing [57], soybean milk fermentation [39,58,59], and the inhibition of acute gastroenteritis [60]. Therefore, the above evidence suggests that SCFAs play an important role in regulating the GI microbiome and human health.

The gut microbiome synthesizes neurochemical substances, thereby affecting the CNS. Dysfunction of the microbiome–gut–brain axis has a crucial impact on neurodevelopmental diseases, such as depression, autism, and AD [61]. Gamma-aminobutyric acid (GABA) is a neuroactive substance involved in neurodegenerative diseases [62]. It can affect the neuropathology of the brain through the microbiome–gut–brain. Fujii et al. [63] established the germ-free C57BL/6N mice with fecal samples from a healthy participant and from an AD patient. Recent evidence suggested that alterations in gut microbiome composition contribute to cognitive decline in AD patients and experimental mouse models [63]. In Fujii et al.’s study, oral transplantation of gut microbiota from an AD patient induced cognitive impairment in recipient mice [63]. The result further demonstrates that compositional variations in intestinal microbiomes lead to alterations in gut microbiome metabolites, which in turn induce distinct behavioral outcomes, thereby supporting the concept of GBA. Additionally, through the comparative analysis of 30 significant differential metabolites, it was observed that levels of GABA, taurine, tryptophan, tyrosine, and valine were elevated in the group transplanted with microbiomes from healthy donors. In contrast, propionic acid was found to be present at lower concentrations [63]. This indicates that the intestinal microbiome can determine the metabolism of specific products, thus affecting the progression of related diseases. The trimethylamine content produced by the uptake of meat and dairy products depends on the gut microbiome situation [29]. However, trimethylamine is oxidized to the harmful trimethylamine N-oxide (TMAO) in the liver. TMAO concentration is positively correlated with the risk of cardiovascular disease [64]. Notably, Vogt et al. detected the level of TMAO in the cerebrospinal fluid (CSF) of individuals with AD clinical symptoms [65]. The results showed that CSF TMAO in subjects with MCI and AD dementia was significantly higher than that of healthy participants, and the enhancement of CSF TMAO level was related to the Tau protein phosphorylation. High dietary fiber intake produces indole propionic acid in the intestine, which can reduce the risk of type 2 diabetes [66] and has potential neuroprotective effects in the treatment of neurodegenerative diseases [67].

### 3.3. Dietary and Aging Factors Affecting Both the Gut Microbiome and AD

Healthy or unhealthy dietary habits are important factors in gut microbiome alteration. Changes in diet increase or decrease some species of microorganisms and thus regulate a series of metabolic processes [41]. Dietary fiber intake has many benefits for maintaining the functionality of the intestinal mucosal barrier [68], regulating bile acid and cholesterol metabolism [49], and helping patients with type 2 diabetes control blood sugar [51]. These advantages are because the gut microbiome can produce SCFAs through the fermentation of dietary fiber, which plays an important role in subsequent metabolism. Notably, SFCAs can cross the BBB to elicit an anti-inflammatory response, consequently preventing neuronal cell death [69]. The composition of the intestinal microbiome between patients with AD and healthy individuals was previously compared. The microbial diversity of patients with AD is significantly lower than that of the control group, and the difference in abundance levels was related to biomarkers of AD [70,71,72]. In contrast, the excessive intake of a diet rich in protein and fat leads to an increase in *Bilophila*, *Alistipes*, and *Bacteroides* in the intestine because they belong to the bile-tolerant categories [73]. This labile gut microbiome composition increases the TMAO concentration in the liver and blood [29], triggers inflammatory reactions and oxidative stress, and eventually develops into various metabolic diseases [35]. Importantly, TMAO can also activate microglia, destroying the integrity of the gut barrier and BBB, leading to neuroinflammation [67]. Wang et al. [74] found that *L. plantarum* significantly reduced the TMAO level in APP/PS1 mice by reshaping the gut microbiome composition and improved pathological features related to AD (cognitive impairment and Aβ plaque).

The number of species of gut microbiome is relatively small at birth but gradually increases over time. The intestine of healthy adults is dominated by *Bacteroidetes* and *Firmicutes*, followed by a small number of *Actinobacteria*, *Verrucomicrobia*, and *Proteobacteria* [75]. However, in the elderly (>65 years old), taxonomic diversity significantly decreases, showing a lower abundance of *Bifidobacterium* and *Bacteroides* than in healthy adults [30]. Most importantly, these two strains have many benefits for normal metabolism and the health of the human body. Moreover, the aging gut microbiome weakens its ability to ferment and decompose substances, thereby producing fewer SCFA and other intestinal metabolites [76]. These changes may be related to a decrease in food intake diversity, changes in living habits, and frailty of the physiological state. The elderly population shows poor dental function and saliva secretion; decreased efficiency of digestion, absorption, and metabolism; lack of exercise; and frequent drug use [43]. Notably, AD prevalence rates also significantly increase with age, doubling approximately every 5 years after the age of 65. Approximately 10% of individuals over the age of 65 years and 30–50% of those over the age of 85 years may develop AD [77]. However, normal aging involves slight cognitive decline; therefore, it is usually difficult to distinguish cognitive decline caused by AD from that related to normal aging [78]. Mild cognitive impairment (MCI) is the earliest transitional stage before the onset of AD. According to Tahami’s summary of PubMed literature in North America, Europe, and Asia from 2014 to 2021, the probability of patients with MCI developing AD ranges from 40% to 75% [1]. It is difficult to prevent or intervene in time during the stage of MCI. Under these circumstances, AD appears to be an inevitable physiological phenomenon of aging. However, the treatment strategy for AD should be reappraised and explored from a more promising perspective to avoid misfortune.

Regardless of the pathogenesis or risk factors, AD may be more like a complex metabolic syndrome than a traditional disease. The occurrence and progression of AD are caused by diverse metabolic mechanisms and environmental risk factors, accompanied by different clinical manifestations [4,79]. This suggests that the ultimate clinical manifestation of AD may involve an assembly of pathogenic pathways. Notably, as key factors related to both AD and gut microbiome, aging and diet not only alter the gut microbial population, leading to GI disturbance and attack on the immune system, but also cause CNS disorders.

## 4. Gut Microbiome in AD

### 4.1. The Gut–Brain Axis

**Figure 2:**
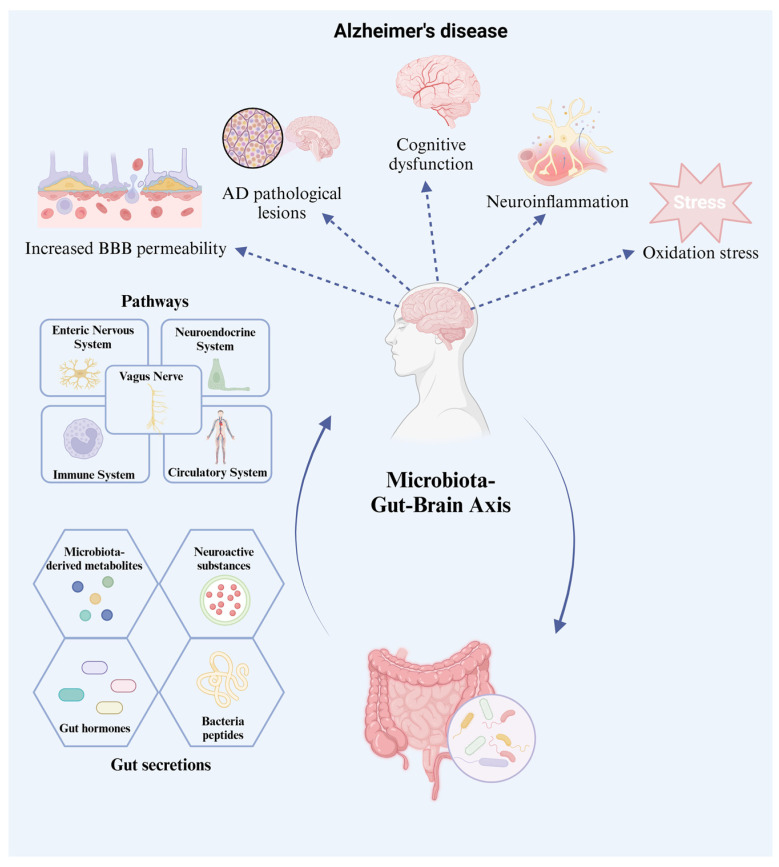
Microbiome–gut–brain axis mediates the translocation of gut secretions into the central nervous system, influencing AD pathogenesis. AD, Alzheimer’s disease; BBB, blood–brain barrier.

**Figure 3:**
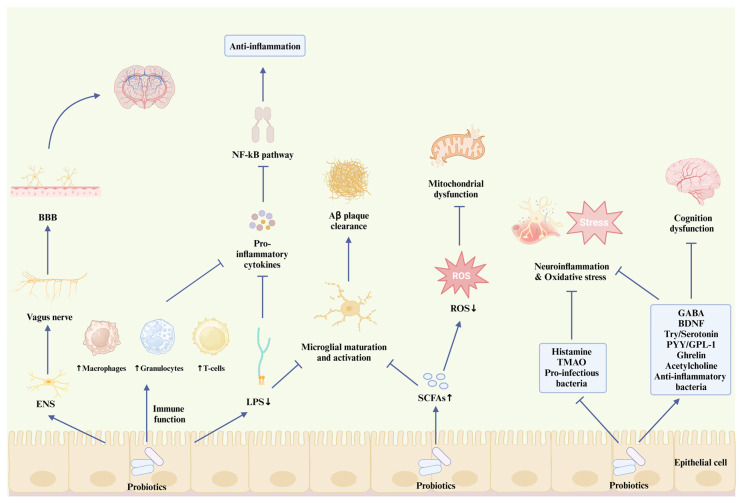
The therapeutic pathways of probiotics in AD pathogenesis. BBB, blood–brain barrier; ENS, enteric nervous system; LPS, liposaccharide; SCFA, short-chain fatty acid; ROS, reactive oxygen species; TMAO, trimethylamine N-oxide; GABA, gamma-aminobutyric acid; BDNF, brain-derived neurotrophic factor; PYY, peptide YY; GPL-1, glucagon-like peptide-1.

The gut microbiome plays an important role in nervous system-related diseases, including neurodegenerative diseases, anxiety, depression, and autism [61]. Dietary habits, lifestyle, xenobiotics, autoimmune conditions, biochemical metabolism, inflammatory reactions, and diseases can influence the composition of the intestinal microbiome [3,80]. Disturbances in the gut microbiome change the ratio of intestinal derivatives, inappropriate neurochemicals, and neuroactive substances produced to act on the CNS in the brain. These intestinal products trigger neuroinflammatory reactions and oxidative stress, leading to an increase in intestinal and BBB permeability, consequently promoting neuronal injury and death. This pathway relies on multiple communication networks [61] in the GBA. Multidirectional contact between the gut and the brain occurs in various ways. Intestinal products, such as microbiome-derived metabolites, hormones, and neuroactive molecules, can regulate the ENS, CNS, neuroendocrine system, and immune system [81,82]. The intestine has an independent neural network; it can function normally even if it is disconnected from the CNS. However, the bottom-up connection between the gut and CNS mainly depends on the vagus nerve in the parasympathetic nervous system, which is composed of afferent and efferent limbs [62]. Metabolites of gut microorganisms reach the brain through the vagus nerve and the circulatory system [81]. This process is of great significance in improving the imbalance between neuroactive substances and other metabolic substances in neurodegenerative diseases. The relationship between the gut and brain is shown in Figure 2. In Bercik’s experiment [83], an anxious BALB/c mouse model (a more timid mouse strain) and an NIH Swiss mouse model were constructed. When the gut microbiome of NIH Swiss mice colonized the gut of BALB/c mice, the levels of brain-derived neurotrophic factor (BDNF) in the hippocampus significantly increased. In contrast, NIH Swiss mice colonized with the BALB/c mouse microbiome no longer showed active exploratory behavior. BDNF is a neuroactive substance regulator that promotes the growth, maturation, and survival of neurons and is related to changing anxiety states [84]. Many gut metabolites affect nervous system diseases via the GBA (Figure 3).

### 4.2. AD Pathogenesis: The Gut Microbiome Effect

#### 4.2.1. Neuroactive Substances

Low BDNF levels in the brain and serum are biomarkers of cognitive dysfunction in AD [85]. BDNF regulates the Ras/MAPK/ERK and IRS-1/PI3K/AKT pathways by binding to tyrosine kinase (TrKB) receptors, thus controlling neuronal survival and death of neurons [86]. BDNF not only participates in the survival of neurons but also activates other intracellular pathways. The ligand (BDNF), activated by tyrosine residues, can regulate the cAMP-response element binding protein (CREB) and CREB-binding protein (CBP), thus affecting neuroplasticity and neuronal resistance [87]. Therefore, BDNF can improve cognitive impairment, learning ability, and memory loss related to the pathophysiology of AD. In a previous study [88], BDNF was injected into adult rat retinal ganglion cells, and the TrkB, MAPK, and PI3K-PKB pathways were activated, thus inhibiting apoptosis induced by caspase-3. After continuous injection of BDNF for 12 days, the percentage of surviving neurons significantly increased. Recently, an increasing number of studies have pointed out the therapeutic potential of BDNF in cognitive dysfunction in neurodegenerative diseases, such as AD [89]. In addition, intestinal bacteria can produce a variety of neuroactive substances such as GABA, histamine, norepinephrine, and serotonin. These neuronal stimulation inducers control synaptic signaling and neuronal function through GBA, thereby influencing cognitive, memory, and learning abilities. The experimental results of Bai et. al. [90] showed that the level of GABA significantly decreased in the parietal lobe of patients with AD, compared with that in healthy participants. *Lactobacillus* and *Bifidobacterium* are the two main intestinal microbiomes that produce GABA [91], and the absence of these two strains in the GI tract may be the reason for lower GABA levels in patients with AD. In contrast, high histamine levels have been associated with ferroptosis and neuroinflammation in AD [12]. *Lactococcus* and *Lactobacillus* in the intestine convert L-histidine into histamine through histidine decarboxylase, thereby increasing the NO level [92], which eventually leads to neuroinflammation and oxidative stress. Tryptophan (Trp) is an essential amino acid involved in three metabolic pathways (kynurenine, serotonin, and aryl hydrocarbon receptor [AhR] pathways) during the decomposition of gut bacteria, which are associated with cognitive ability in AD [93]. In the serotonin biosynthesis pathway, the intermediate products, circulating Trp or 5-hydroxytryptophan (5-HTP), can directly reach the brain and produce serotonin in the CNS [94]. Serum can improve the clinical symptoms and pathogenesis of AD [95,96,97]. Indole derivatives exerted similar neuroprotective effects. They are ligands of AhR and can inhibit neuroinflammatory reactions by activating the AhR signaling pathway [67].

#### 4.2.2. Microbiome-Derived Metabolites

In addition to neurotrophic factors and neuroactive substances, microbiome-derived metabolites such as SCFAs, LPS, and TMAO are key factors associated with the pathological manifestations of AD. SCFAs are the most common metabolites derived from the gut microorganisms. Presently, most studies have shown that SCFA mainly antagonizes AD in three ways: through the anti-inflammatory reaction, reducing Tau protein phosphorylation caused by Aβ plaque, and increasing neuroactive substances to improve memory and cognition [79,93]. SCFA can promote the secretion of anti-inflammatory factors through the immune system, thus inhibiting neuroinflammation. SCFA can enhance the ability of microglia to clear Aβ of microglia and prevent excessive phosphorylation of Tau protein. However, LPS and TMAO will promote amyloids to pass through the BBB, leading to the Aβ aggregation in the brain and activation of microglia, triggering the neuroinflammatory reaction. However, butyric acid can decrease the levels of LPS and inhibit the activation of microglia and secretion of pro-inflammatory cytokines through the anti-inflammatory pathway [72].

#### 4.2.3. Ghrelin Hormone

Ghrelin is a hormone released from the stomach during hunger and enters the brain through the BBB, thus stimulating appetite. It regulates food intake by binding to the growth hormone secretariat-receptor (GHS-R) [98]. Moreover, this functional hormone can regulate a variety of metabolic activities, such as muscle homeostasis and insulin sensitivity neuromodulation [99]. Deacylated ghrelin, the most abundant form in the circulatory system, is converted into acylated ghrelin (AG) by ghrelin O-acyltransferase (GOAT) [100]. Only AG is a corresponding ligand of the GHS-R1a receptor. The combination of AG and GHS-R1a receptors in the nucleus of the hypothalamus initiates the synaptic transmission of neurons, thus helping to improve cognitive function and memory ability [101]. An AD clinical report by Gahete et al. [102] found that the expression level of GHS-R1a in the temporal lobe was significantly lower than that in the healthy control group. In Moon et al.’s report [103], high-dose ghrelin supplementation significantly improved hippocampal function and neuroinflammation. However, Cao et al. [104] showed that the level of AG in the brains of patients with AD was higher than that in the control group. This result seems to conflict with the neuroprotective effect of ghrelin, which may be interpreted as a compensatory response due to a severe deficiency of the ghrelin receptor. High-dose ghrelin plays a key role in improving AD-related pathogenesis.

## 5. The Interaction of Probiotics with GBA and Its Significance in AD

### 5.1. Functional Properties of Probiotics

Probiotics are non-pathogenic live microorganisms consisting of yeast and diverse beneficial bacteria. Appropriate intake of probiotics can maintain the balance of the gut microbiome and regulate the metabolic cycle. This is because of several characteristics of probiotics. They must tolerate the decomposition of acids, bile, and pancreatic juice to survive, colonize, and reproduce in the GI tract [105]. Probiotics have a strong competitive ability to limit the quantity of nutrients by pH adjustment in the intestine, thus invoking antagonism against harmful bacteria [106]. These nutrients are energy sources for the intestinal epithelial cells and various bacteria. Probiotics can repair intestinal epithelial cells, maintain the integrity of the gut mucosa, and establish a gut barrier to protect the host immune system [107]. Probiotics also play a crucial role in regulating bile metabolism and increasing bone density and have anti-allergic and anti-inflammatory properties [106]. Regarding bile acid metabolism modulation, *Bifidobacterium* species can secrete bile salt hydrolase (BSH), which deconjugates primary bile acids into secondary forms, activating the farnesoid X receptor (FXR) to enhance enterohepatic circulation [108]. This has also been proven in Hu et al.’s study: *Lactobacillus rhamnosus* GG-administered mice exhibited a significant reduction in unconjugated bile acid excretion and an increase in BSH levels [109]. In addition, probiotics can prevent and improve many diseases, such as GI diseases (IBD), liver disease, genitourinary infections, metabolic diseases (obesity, diabetes, and cardiovascular disease), immune-related diseases, cancer, oncologic diseases, and neurological diseases (anxiety, depression, autistic spectrum disorder, and neurodegenerative disease) [43,105]. For example, *Lactic acid bacteria* (LAB) (such as *Bifidobacteria* and *Lactobacilli* strains) can modulate gut microbiome composition to indirectly influence SCFA levels, with butyrate, produced by *Firmicutes phylum* families, demonstrating pro-apoptotic and anti-proliferative effects on colorectal cancer cells [110]. Furthermore, LAB exhibits anti-carcinogenic potential via immune regulation (such as cytokine modulation and phagocyte activation) and demonstrates therapeutic efficacy against human papillomavirus (HPV)-related cervical neoplasia [111]. Importantly, probiotics regulate intestinal movement and metabolism through the ENS and access the CNS via a bidirectional contact pathway [11]. Probiotics affect AD via the gut–brain interaction network. They control the release of neuroactive substances by modifying the composition of the gut microbes, thereby facilitating the survival and differentiation [86]. In addition, they can directly adjust brain biochemical components, such as serotonin [83] and BDNF [84], in the brains of AD mouse models to treat cognitive and memory deficits. For instance, comparative analysis by Bai et al. revealed that parietal lobe GABA levels in AD patients were attenuated by approximately 40% compared to neurologically intact participants [90]. This neuroregulatory capacity originates from microbial metabolites, particularly SCFAs, where butyrate enhances tryptophan hydroxylase activity in enterochromaffin cells to promote 5-HT precursor synthesis [94].

### 5.2. Commonly Used Probiotics

Currently, the most widely used probiotics are *Lactobacillus* and *Bifidobacterium*, which have been declared safe and beneficial to the human body [112]. *Lactobacillus* is a genus of gram-positive facultative anaerobic rods or microaerophilic rod-shaped non-spore-forming bacteria, including *Lactobacillus plantaru*, *Lactobacillus brevis*, *L. casei*, *L. acidophilus*, and *L. fermentum* [113]. They produce acidic environments by converting hexose sugars into lactic acid, thus inhibiting the growth of various harmful bacteria [114]. The *Bifidobacterium* genus consists of gram-positive non-motile anaerobic bacteria, including *Bifidobacterium breve*, *Bifidobacterium longum*, *B. infantis*, and *B. animalis subsp lactis* [113]. They not only survive in gastric acid, bile salts, and other biological body fluids but also play a beneficial role in human health. Nimgampalle et al. [115] pointed out that a 60-day treatment with *L. plantarum* significantly improved cognitive impairment and restored the level of acetylcholine in AD rats (a D-galactose-induced AD model). Amyloid aggregation increases acetylcholinesterase expression, leading to a decrease in acetylcholine levels and cognitive and memory abilities. Probiotics can increase acetylcholine levels, thus improving the symptoms of cognitive impairment and spatial memory loss. Lee et al. [116] isolated *B. longum* from human intestines. It can inhibit the activation of inflammatory factors, relieve the imbalance of gut microbiome in AD model (5xFAD transgenic mice) and aged mice, and improve AD-related symptoms, such as cognitive dysfunction. Athari Nik Azm et al. [117] treated the AD group (Aβ1-42 injection rats) with *Lactobacillus* (*L. acidophilus* and *L. fermentum*) and *Bifidobacterium (B. lactis*, *B and longum*) for 8 weeks. The results of the water maze test showed that the spatial memory ability of the probiotic group significantly improved compared with that of the AD group. Therefore, both single and mixed probiotics are of great significance in improving AD. Additionally, probiotics can enhance the therapeutic effects of AD drugs. Wang et al. [74] showed that the combination of memantine (a medicine for dementia) and *L. plantarum* for 12 weeks reduced the levels of Aβ1-42 and Aβ1-40 in the hippocampus of APP/PS1 mice to a greater extent, compared to that with the memantine treatment alone. Combining probiotics with other interventions may play a substantial role in the treatment of AD.

### 5.3. Prebiotics and Symbiotics

The scope of research on AD probiotics is expanding gradually. Symbiotics combine the synergistic effects of probiotics and prebiotics. Prebiotics are fiber-based compounds that tolerate gastric acid and are not decomposed by digestive enzymes [118]. Prebiotics include cellulose, lignin, oligosaccharides, raw oats, and soybeans [68]. Probiotics ferment and decompose prebiotics into nutrients, thereby promoting SCFA secretion and enhancing host immunity [119]. Both probiotics and prebiotics are beneficial for AD treatment, and symbiotics combine their respective beneficial characteristics to achieve a compelling therapeutic effect. Fruits and vegetables are rich in prebiotics, such as inulin, fructooligosaccharides (FOS), and xylooligosaccharides (XOS), which are the main forms of fructan [120]. These dietary fibers can promote the colonization and growth of probiotics, such as *Bifidobacterium,* in the intestine. In addition, they reduced neuroinflammation and downregulated AD biomarker levels. Chen et al. [121] found that FOS effectively improved the cognitive function of AD mice and downregulated the overexpression of Aβ1-42 and Tau protein. FOS also showed potential as a prebiotic by increasing the abundance of *Bifidobacterium* and *Lactobacillus*. In the results of anti-inflammatory effect, all anti-inflammatory indexes have recovered to the baseline after the treatment of FOS and *Bifidobacterium*, and the treatment effect of the symbiotic group (FOS + *Bifidobacterium*) is the most significant.

### 5.4. How Probiotics Interact with AD Pathogenesis

#### 5.4.1. Probiotics and Aβ Plaque

The gut microbiome is involved in the pathogenesis of AD through various biochemical metabolic pathways. Bacterial amyloid has a similar molecular metabolism pattern to β-amyloid, evoking Aβ misfolding and aggregation [122]. The disordered intestinal environment gives improper proteins the chance to easily pass through the BBB via the GBA [123], resulting in excessive deposition of amyloid in the brain. This occurs in the early stages of MCI in AD and is one of the most important pathogeneses of AD. Moreover, the destruction of the intestinal barrier and changes in BBB permeability stimulate the inflammatory signaling pathway, accelerate the injury of neural cells by inflammatory factors, and ultimately kill neurons [124].

Various species of microorganisms in the human gut can produce a marked number of LPS, amyloids, and decomposition products. These secreted substances are usually quite soluble; thus, tissues and systems have a certain tolerance [125]. However, under long-term exposure to the burden of metabolism, they gradually develop into highly insoluble fibrous protein aggregates, leading to the occurrence of neurodegenerative diseases characterized by amyloid accumulation [126]. This phenomenon has been observed in brain samples of patients with AD by proteomic analysis of amyloidogenic fungal proteins [127]. Asti and Gioglio [128] pointed out that after long-term injection of bacterial LPS into the brain of rats, several pathological characteristics, similar to those in the brain of patients with AD, occurred. Insoluble fibrous protein aggregates cause damage to lipid membranes and neurotoxicity [129] and have been found on the surface of fungi and bacteria (such as *Escherichia coli*, *Bacillus*, *Staphylococcus*, and other gram-negative bacteria that usually trigger human diseases) [122,125,128,130]. Bacteria utilize amyloid proteins as structural materials of bacterial amyloid fibers to promote adhesion and the recruitment of pathological protein assemblies, eventually destroying the intestinal barrier and attacking the host immune system [129]. Curli is the first bacterial amyloid fiber discovered by Chapman et al. [131], which has a similar structure and function to the Aβ protein [132] because they aggregate in the same way as Aβ peptides. Bacterial amyloid has the same pathogen-associated molecular patterns as Aβ 42, which can be recognized by toll-like receptors (TLRs) and then trigger an immune response and neuroinflammation [125]. Enteroendocrine and other epidermal cells can allow the misfolded α-synuclein deposit to enter the brain through the direct neural connection of alpha-synuclein, leading to the Aβ aggregation in the CNS [131].

Probiotics resist the deposition of Aβ plaque in two ways: the modulation of gut metabolites and the regulation of gut microbiome composition. Probiotics can increase the level of SCFAs to interfere with protein–protein aggregation, thus eliminating the formation of Aβ plaque (Figure 3). Kobayashi et al. [133] found that the administration of *Bifidobacterium brevis* A1 not only downregulated the gene expression of hippocampal immune response induced by Aβ but also increased the level of acetate in the plasma of mice. However, Kobayashi et al. did not analyze the inhibitory effect of *B. brevistrain* A1 on clearing Aβ accumulation. The Aβ clearance effect of probiotics was verified in the in vitro study by Yeon et al. [134]. They found that *Lactobacillus helveticus* IDCC 3801 (in fermented milk) has the potential to reduce the level of Aβ and improve APP metabolism. In addition to micro-biota-derived metabolites, such as SCFAs, neuroactive substances, such as serotonin, have a similar effect on alleviating AD intensity by participating in Aβ plaque clearance. Cirrito et al. [96] showed that after long-term administration of selective serotonin reuptake inhibitors (SSRIs), the plaque load in the brains of mice decreased by 50%. They also analyzed the brain amyloid load of healthy elderly participants and those who had used SSRIs in the past five years. The results showed that serotonin signaling was related to the reduction in amyloid plaques. Serotonin is involved in the α-secretase and β-secretase cleavage pathways of APP proteolytic metabolism [97]. The serotonin receptor is activated to initiate the extracellular signal-regulated kinase (ERK) pathway. Phosphorylated ERK will increase the cleavage effect of α-secretase on APP while inhibiting the activity of β-secretase [96]. Probiotics can inhibit the accumulation of Aβ plaque by participating in the metabolism of 5-HT and play a positive role in mitigating the pathological intensity of AD [94,95]. In addition, probiotics can maintain the balance of the gut microbiome and improve AD-related symptoms, such as amyloid plaque deposition, by increasing the beneficial microbiome and suppressing antagonistic pathogens (Figure 3). Cattaneo et al. [135] explored the relationship between amyloid content and the composition of the gut microbiome. Compared with the control group, the abundance of the anti-infectious EU bacteria rectum was lower, while that of pro-infectious *Escherichia* or *Shigella* was higher in the feces of the Aβ deposition and cognitive impairment groups. Proinflammatory bacteria can cause α-synuclein misfolding, thus allowing more bacterial amyloids to enter the brain through the BBB. Bacterial endotoxins may play an important role in the formation of amyloid plaques and other pathological processes related to AD. Wang et al. [136] found that the administration of *L. plantarum* MA2 reshaped the gut microbiome composition of AD rats (D-Galactose/AlCl3 induced AD model) and inhibited the aggregation of Aβ_42_ and the amyloid-induced cytotoxicity. Probiotics maintain the intestinal immune system by competing for limited nutrition and releasing anti-bacterial substances, consequently playing an antagonistic role in the pathological processes related to AD. Hence, metabolites released by a healthy intestinal microbiome can regulate immune function, reduce Aβ accumulation, and mediate the inflammatory reaction against AD. In contrast, the disorder of the gut microbiome will aggravate the inflammatory reaction and oxidative stress of the CNS, leading to the deposition of Aβ.

#### 5.4.2. Probiotics and Neuroinflammation

Several bacterial strains can be involved in the formation of Aβ fibrils and Tau phosphorylation, such as *E. coli*, *Salmonella enterica*, and *Staphylococcus aureus* [137]. Excessive accumulation of Aβ plaque in the brain will accelerate the phosphorylation of Tau protein, leading to an inflammatory reaction. Intestinal epithelial cells recognize different antigens carried by or around the surface of the gut microorganisms [138]. LPS is connected to the outer membrane of gram-negative bacteria, which mediates the association between the imbalance of the intestinal microbiome and the pathological process of Tau protein in AD [139]. From the perspective of the neuronal pathway, LPS causes abnormal activation of microglia, which decreases their clearance capacity for Tau protein and Aβ plaque, thus causing neuroinflammation (Figure 3). The hyper-ramified (a type of activated microglia) [140] and dystrophic microglia [141] have been found in the postmortem brains of patients with AD. These modified microglia no longer exert protective effects against neuroinflammation. The combination probiotic treatment with *B. lactis*, *L. brevis*, and *L. fermentum* decreases the number and modification of microglia in the hippocampus and cerebral cortex of mice (5xFAD model) [142]. The therapeutic effect of probiotics significantly reduced the levels of tau protein aggregation and phosphorylation in the 5xFAD group, which was comparable to the therapeutic effect of phosphatidylserine (a protective component of the nerve cell membrane). Probiotics may alleviate pathological processes related to AD by inhibiting microglial activation (Figure 3). In addition, from the perspective of immunological signaling pathways, LPS is allowed to cross the BBB through interaction and recognition with the constitutively expressed CNS receptors, CD14 and TLR4, triggering the corresponding immune response. TLR4 phosphorylates the MyD88 pathway to activate the pro-inflammatory transcription factor, NF-κB [139,143]. The activation of this pathway promotes macrophage activation and the release of pro-inflammatory cytokines [144]. Probiotics can also regulate immune functions to maintain intestinal homeostasis and fight this invasion (Figure 3). Probiotics can inhibit the signaling pathways of pro-inflammatory cytokines by affecting the distribution and function of immune cells (macrophages, granulocytes, and T-cells) [145]. Jeong et al. [146] showed that 8 weeks of *Lactobacillus pentosus var. plantarum C29* treatment inhibited the expression of the NF-κB signaling pathway of aging rats (Fischer 344 model) and aging-induced pro-inflammatory factors, TNF-α and IL-6.

#### 5.4.3. Probiotics and Mitochondrial Function

Mitochondrial dysfunction occurs in the early pathological stage of AD and is associated with the accumulation of NFT induced by amyloid plaques and tau protein phosphorylation [147]. Excessive accumulation of these toxic proteins and fibers produces ROS, which will lead to the aggravation of oxidative stress. Importantly, brain neurons are high-energy demanders in human tissues and organs, accounting for 20% of the oxygen consumption in the entire body, even under resting conditions [148]. Therefore, a continuous energy supply is the basis for neurons to maintain a steady state and function. Oxidative stress can cause serious damage to neurons and mitochondria, leading to neuronal death. Mitochondria are mediators in iron metabolism and Ca^2+^ homeostasis, including heme protein synthesis, redox signaling, energy metabolism, and neuroactive substance transmission [28,147,149]. However, excessive ROS formation leads to the uncontrolled regulation of iron. This interrupts the regulation and control of related proteins and cofactors in iron metabolism, leading to iron imbalance, mitochondrial energy deadlock, and even iron death [148]. Ferroptosis is an iron-dependent form of nonapoptotic cell death [12] that is induced by lipid peroxidation when ROS are overloaded [26]. The severity of the iron load in the hippocampus and thalamus of patients with AD was directly proportional to a decline in cognitive ability [150]. In addition, Van Bergen et al. [151] scanned and evaluated the iron load and Aβ plaque level of 116 elderly participants with quantitative susceptibility mapping and 18F-Flutemetamol-PET, respectively. There was a significant correlation between the increase in iron and Aβ accumulation (*p* < 0.05). However, amyloid deposition and tau protein phosphorylation may also lead to calcium ion disorders and mitochondrial dysfunction by disrupting the calcium ion circuitry signaling pathway in neurons [152]. Pérez et al. [153] found mitochondrial damage, decreased ATP levels, and Ca^2+^ overload in the peripheral tissues of patients with AD.

Notably, mitochondria and the ancient intestinal micromicrobiome (both anaerobic and aerobic bacteria) have a common development history [154]. This interaction may explain why an intestinal microbiome imbalance affects mitochondrial function [155]. Probiotic interventions can mitigate mitochondrial oxidative stress by reducing ROS levels, thereby improving the pathology of AD (Figure 3). *Acidophilus* improved mitochondrial dysfunction by reducing the levels of oxidative stress biomarkers, thus alleviating cognitive dysfunction in AD rats (D-gal- and ALCL3-induced models) [156]. A similar result was found in the report of Ton et al. [157]. Kefir-fermented milk treatment for 90 days significantly reduced the magnitude of serum protein oxidation in patients with AD and improved mitochondrial dysfunction and cognitive defects. Probiotics, including *Lactobacillus* [158,159] and *B. lactis* [160], not only have a natural antioxidant defense system but can also activate antioxidant enzymes (superoxide dismutase, catalase, ascorbate peroxidase) in the host. Probiotics are also of great importance in the inhibition of iron and calcium ion disorders. Probiotics inhibit the Fenton reaction during ferroptosis by producing catalase, thus establishing an antioxidative defense mechanism in neural cells [161]. *Lactobacillus casei* can chelate Fe^2+^ to inhibit lipid peroxidation [162]. *Lactococcus lactis* MG1363-pMG36e-GLP-1, a probiotic strain constructed by Yue et al. [163], reduces oxidative stress indices, such as ROS and malondialdehyde, and effectively suppresses ferroptosis by activating the Keap1/Nrf2/GPX4 signaling pathway. They also found that synthetic probiotics corrected the intestinal microbiome composition in mice: the levels of *Akkermansia*, *Oscillospira,* and *Sutterella* increased. Although this study focused on the neuroprotective effect against Parkinson’s disease, the evaluation results (iron death caused by mitochondrial damage, oxidative stress, and alteration of intestinal microbiome) are also related to the pathological mechanism of AD, which provides a theoretical basis and database for future treatment strategies for AD. Additionally, the experimental results of Sobol et al. [164] have shown that products fermented by *Lactobacillus* regulate nerve signal conduction by adjusting calcium homeostasis in nerve cells.

#### 5.4.4. Probiotics and Other AD Pathological Hypotheses

The etiology of AD remains unclear. Apart from the main AD pathology mentioned above, multiple pathogenesis hypotheses have been proposed to understand the specific causes or mechanisms of AD. The cholinergic hypothesis proposed that the decrease in acetylcholine level (the main neuroactive substance of cholinergic neurons) induces Aβ accumulation and Tau protein abnormality, eventually leading to oxidation stress and neuronal cell apoptosis [28]. Cholinergic neurons in the basal forebrain (BFCN) regulate advanced behaviors, such as cognition, learning, and memory. Therefore, the degeneration of the BFCN in patients with AD leads to the loss of synapses between the basal forebrain and the corresponding tissues of the cortex and hippocampus, leading to a reduction in cognition, memory, and learning abilities [165]. This has been found in early clinical evidence [166], and the brain tissue evaluation results of patients with dementia exhibited low acetylcholine levels and a lack of cholinergic neurons. Probiotics have the potential to act as acetylcholinesterase inhibitors in cognitive impairment by increasing acetylcholine levels [115,167,168].

From the perspective of epidemiological research, risk factors, such as vascular pathologies, hypertension, and diabetes, are related to cognitive dysfunction and an increased risk of AD [169]. The oxygen consumption of the brain accounts for approximately 20% of the total oxygen supply in the whole body and receives 15% of the cardiac blood output. Thus, damage to cerebral perfusion can easily induce heart failure and vascular pathological changes [170]. According to the vascular hypothesis of AD, these cardiovascular risk factors cause destruction of the BBB and changes in vascular structure and cerebral hemodynamics, which contribute to insufficient cerebral blood flow and oxygen supply, eventually leading to the development of AD-related pathology [28]. Mitochondria and cholinergic neurons are vulnerable to damage under long-term hypoxia [171]. Clinical evidence has shown that probiotics can reduce the risk of cardiovascular diseases. Probiotics reduce total cholesterol and low-density lipoprotein cholesterol levels, thus alleviating and preventing cardiovascular diseases, such as hypertension [172]. Notably, higher cholesterol levels were found in patients with AD with AD harboring the *ApoE4* gene (which regulates lipid transport and metabolism) [170], which is also the strongest genetic risk factor for AD. Abnormal cholesterol metabolism may induce the formation and accumulation of Aβ plaque [173]. In addition, a meta-analysis by Tao et al. [174] concluded that probiotics can reduce three important indices of patients with type 2 diabetes (fasting blood glucose, glycosylated hemoglobin A1c, and insulin resistance). Tenorio-Jiménez et al. [175] reported that probiotics can significantly reduce the inflammatory index of the obese population, thus reducing the risk of related diseases, such as CVD and diabetes. The pathological mechanism of AD is complicated because it interacts and synergizes with one diseases. Probiotics, by virtue of their various regulatory pathways, participate in every pathology of AD, thus playing a comprehensive and covering improvement role in AD therapy. The probiotic effect associated with AD pathogenesis has been summarized in Figure 3.

## 6. Applications, Prospects, and Limitations of Probiotics

Currently, *Lactobacillus* is the most frequently used genus in probiotic therapy and most studies summarized in this review have shown a positive effect on the pathological process of AD. Generally, probiotics indirectly affect various products secreted by intestinal bacteria by correcting the composition of the intestinal microbiome, thus playing a positive role in many AD pathologies. Recently, combination therapies with probiotics have also been widely studied, including the combination of probiotics with AD drugs [74] and the administration of symbiotics [121]. However, there are several limitations to these positive effects.

### 6.1. Different Intervention Conditions of Probiotcs

Probiotic administration varies and some variables need to be explored through numerous longitudinal experiments. In the existing clinical trials, the treatment schemes for probiotics include single-strain probiotics, mixed probiotics, and combined interventions; the intervention duration is also neglected. The majority of existing clinical investigations on probiotic interventions have adopted relatively short observation periods, typically spanning 2–3 months. As comprehensively reviewed in the report by Naomi et al., even a two-week probiotic intake for animals or four-week probiotic treatment for humans are sufficient to improve the pathology related to AD [176]. Nevertheless, probiotics with therapeutic potential, such as *Lactobacillus* and *Bifidobacterium*, may require extended follow-up periods to fully elucidate the critical assessment of potential microbiome adaptation and metabolic homeostasis maintenance. Current research gaps highlight the imperative for longitudinal studies incorporating repeated outcome assessments, standardized dosing protocols, and comprehensive evaluation of microbial viability during prolonged administration. Additionally, probiotics are strain specific and exhibit significant individual variance. This may explain why the same effective probiotics did not achieve the expected improvement after intervention in another study. In a randomized double-blind placebo-controlled trial by Akbari et al. [177], patients with AD were treated with probiotics for 12 weeks. The results showed that although the cognitive function of patients with AD was obviously improved, the biomarkers related to oxidative stress and inflammation did not significantly change. Hsu et al. [178] also treated patients with AD with *Bifidobacterium* and *Lactobacillus* for 12 weeks. The results showed that cognitive impairment, oxidative stress, and inflammatory responses significantly improved. This positive improvement is similar to the results of Hsu et al. [178] but may be attributed to the combined effect of selenium supplementation and probiotics. Kobayashi et al. [179] investigated the effect of a 12 week-treatment with *B. brevis* A1 on the prevention of cognitive and memory improvement in subjects with MCI. The results showed that not all cognitive ability test scores were significantly different, which implies that the therapeutic effect of single-strain probiotics requires further research to be verified. Moreover, the participants reported by Tini et al. [170] were all patients with MCI. This indicated that the severity and progression of AD may also affect the efficiency of probiotic interventions. The various intervention conditions for probiotics are compared in Table 1.

### 6.2. The Complexity in Designing an Effective Probiotic-Based Intervention for AD

Currently, most studies are based on in vitro culture and animal models. Moreover, there are many ways to induce an experimental mouse model; therefore, the composition of the intestinal microbiome will be affected differently after induction. Although the induction occurred in a specific direction, it also led to deviations in the experiment. These deviations were amplified after shifting from animal experiments to clinical treatments. In addition to the complexity of the pathological mechanism of AD, genetic factors, dietary habits, emotional changes after intervention, sex of the subjects, and different cognitive evaluation tools in the experiment lead to deviations in the experimental results [180]. Most pathological studies related to AD can only be conducted after death; therefore, there is little pathological evidence related to AD. Additionally, the severity of AD may affect the intestinal response to probiotic interventions. Agahi et al. [181] treated patients with AD with probiotics for 12 weeks and evaluated the severity of AD in patients using Test Your Memory (a cognitive assessment tool). The results showed that 83.5% of the subjects developed serious AD, and no significant changes were found in cognitive scores or other inflammatory and oxidative indices after intervention with probiotics. The development of AD may affect the sensitivity of patients to probiotic therapy. This may be because the loss of neurons and TNFs is an irreversible pathological change in AD [182]. Probiotics may no longer reverse the pathological process in the late stages of AD, based on Agahi et al. [181]. Moreover, it is difficult to distinguish MCI from the normal physiological phenomena of aging. Therefore, it is necessary to apply interventional therapy in the early stage of AD. Hwang et al. [183] reported that a 12-week probiotic supplement of *L. plantarum* C29-fermented soybean significantly improved comprehensive cognitive functions (attention, memory, and language ability) in patients with MCI by stimulating the secretion of BDNF. Previous reports have found that other functional component therapies have a positive impact on the cognitive ability of patients with MCI in short-term interventions (2 weeks or even less). Lin et al. [182] emphasized the importance of early intervention in AD. They found that a four-week treatment of solanezumab (a monoclonal antibody against Aβ plaque) reduced the Aβ accumulation level in the brains of patients with AD, but there was no pronounced change in cognitive ability. Although the complex pathological mechanisms of AD, such as the aggregation of amyloid plaques, are still unclear, all the pathological mechanisms of AD are related. The origin of all these progressions of AD is the deposition of Aβ in the brain of patients with AD. Therefore, an extensive process to explore these complex processes and a comprehensive treatment method are necessary to intervene in AD. The clinical traits revealing the role of probiotics in AD pathogenesis are summarized in Table 1.

## 7. Conclusions

Evidence based on studies in this review illustrates that probiotics can regulate the stability of the intestinal microbiome and secrete intestinal metabolites and neuroactive substances, thus suppressing AD-related pathogenesis, including the increase in BBB permeability, Aβ accumulation, cognition dysfunction, neuroinflammation, and oxidative stress. To date, no drugs have been developed to prevent AD symptoms. In addition, the multifactorial nature of AD pathogenesis, characterized by diverse etiological hypotheses and modifiable risk factors, presents significant challenges for therapeutic development. However, probiotic-based interventions may modulate multiple AD-associated pathways through GBA, demonstrating potential for attenuating neurodegenerative processes. This therapeutic paradigm aligns with evolutionary biology principles—as symbiotic partners in human evolution, gut microbiota exerts systemic influences on human health, mood, and even behavior via bidirectional microbiota–gut–brain communication. More clinical studies on the mechanisms of probiotic effects are necessary to position microbiota-targeted strategies as multimodal interventions in AD management protocols.

## Figures and Tables

**Table 1:** Summarizing the experiments and clinical traits revealing probiotics’ effect on AD pathogenesis.

Probiotics	Model or Subjects	Duration	Significant Effect on AD	Source
200 mL/d of fermentum milk contained *Lactobacillus acidophilus*, *L. casei*, *L. fermentum* and *Bifidobacterium bifidum* (2 × 10^9^ CFU/g for each)	AD patients	12 weeks	Cognitive function	Akbari, 2016 [177]
*Bifidobacterium longum* subsp. *Infantis*, *B. breve*, *B. animalis* subsp. *Lactis*, *B. bifidum* and *Lactobacillus plantarum* (1 capsule contained 1 × 10^10^ CFU/d)	AD patients	12 weeks	↑BDNF Inhibiting oxidative stress and inflammation	Hsu, 2023 [178]
Combination treatment of selenium (200 mg/day) and *Lactobacillus acidophilus*, *L. casei*, *L. fermentum* and *Bifidobacterium bifidum* (2 × 10^9^ CFU/g for each)	AD patients	12 weeks	Ameliorating the cognitive function, oxidative stress and inflammation	Akbari, 2016 [177]
*Bifidobacterium brevis* A1 (2 capsules contained > 2.0 × 10^10^ CFU/d)	MCI patients	12 weeks	Improving the immediate memory	Kobayashi, 2019 [179]
2 mL/kg/d fermented milk contained *Acetobacter aceti*, *A.* sp., *Lactobacillus delbrueckii*, *L. fermentum*, *L. fructivorans*, *L. kefiranofaciens*, *Enterococcus faecium*, *Leuconostoc* spp., *Candida famata* and *C. krusei* (probiotics dosage is not mentioned)	AD patients	3 months	Inhibiting the mitochondrial dysfunction, DNA damage and apoptosis Mitigating the oxidative stress and inflammation Improving cognitive deficits	Ton, 2020 [157]
*Bifidobacterium brevis* A1 (probiotics dosage is not mentioned)	Aβ_25–35_-injected mice	10 days	Ameliorating Cognitive function Increasing the expression of the immune response gene ↑plasma acetate levels	Kobayashi, 2017 [133]
*Lactobacillus plantarum* (1 × 10^8^ CFU/kg/d)	D-galactose/AlCl_3_ induced AD rats	12 weeks	Reshaping the gut microbiome composition Ameliorating Cognitive function ↓Aβ accumulation	Wang, 2022 [136]
8 mL sterilized water of *Bifidobacterium lactis*, *Limosilactobacillus fermentum* and *Levilactobacillus brevis* (8 × 10^7^ CFU/d)	5xFAD mice model	3 months	Attenuating the microglial activation ↓Aβ accumulation ↓Tau phosphorylation Modifying the gut microbiome composition Improving the spatial and recognition memories	Kim, 2024 [142]
*Lactobacillus pentosus* var. *plantarum* C29 (2 × 10^9^ CFU/d)	Aged Fischer 344 rats	8 weeks	Inhibiting NF-κB signalling pathway ↓the expression of BDNF Enhancing the aging-impaired memory	Jeong, 2015 [146]
2 g (1 × 10^10^ CFU/g) of *Lactobacillus acidophilus*, *L. fermentum*, *Bifidobacterium lactis* and *B. longum*	Aβ_1-42_-injected mice	8 weeks	Ameliorating memory deficit and oxidative stress Modifying the microbiome composition	Athari Nik Azm, 2018 [117]
Combination treatment of Memantine (1 mg/mL) and *Lactobacillus plantarum* (1 × 10^9^ CFU/mL)	APP/PS1 mice	12 weeks	Potentiating the drug therapy: Inhibiting amyloid plaque and neuroinflammation Protecting neurons and the hippocampal plasticity ↓TMAO	Wang, 2020 [74]

↑, up-regulate; ↓, down-regulate.

## Data Availability

No new data were created or analyzed in this study. Data sharing is not applicable to this article.
